# Prevalence and characterization of pain in patients with Charcot-Marie-Tooth disease type 1A

**DOI:** 10.1590/0004-282X-ANP-2020-0132

**Published:** 2021-05-09

**Authors:** Helen AZEVEDO, Henrique COSTA, Eduardo DAVIDOVICH, Camila PUPE, Osvaldo José Moreira NASCIMENTO

**Affiliations:** 1 Universidade Federal Fluminense, Hospital Universitário Antônio Pedro, Departamento de Neurologia, Niterói RJ, Brasil. Universidade Federal Fluminense Universidade Federal Fluminense Hospital Universitário Antônio Pedro Departamento de Neurologia Niterói RJ Brazil

**Keywords:** Charcot-Marie-Tooth Disease, Pain, Doença de Charcot-Marie-Tooth, Dor

## Abstract

**Background:**

Charcot-Marie-Tooth disease type 1A (CMT1A) is the most common form of hereditary neuropathy.

**Objective:**

To investigate the prevalence and characteristics of pain in patients with CMT1A.

**Methods:**

Nineteen patients with a diagnosis of CMT1A were evaluated between September 2018 and October 2019, and other causes of neuropathy were ruled out. The following tools were used for the pain assessment: neurological assessment, LANSS, DN4, clinical evaluation, VAS, CMTNS2 and SF-36. Statistical analysis was performed using prevalence analysis, t test, chi-square test and Spearman's rho.

**Results:**

The prevalence of pain was 84.2% in the sample of this study, with moderate intensity and nociceptive characteristics according to the LANSS scale (75%) and clinical evaluation (50%), but differing from DN4, which found neuropathic pain in the majority of the patients (56.2%). Mixed pain was also observed in 43.7% of the patients, according to clinical criteria. There was a statistically significant correlation between pain intensity and SF-36, thus demonstrating that the lower the pain was, the lower the impairment was, in all domains.

**Conclusion:**

Pain is a prevalent and important symptom in CMT1A, with moderate intensity and nociceptive characteristics according to two tools, but neuropathic pain is also present, and there may even be a mixed pattern of pain. The correlation of the pain with SF-36 suggests that pain relief could provide improvements to the quality of life of these individuals.

## RESUMO

**Introdução:**

A doença de Charcot-Marie-Tooth tipo 1 A (CMT1A) é a forma mais comum de neuropatia hereditária.

**Objetivo:**

Investigar a prevalência e as características de dor nos pacientes com a doença de CMT1A.

**Métodos:**

Dezenove pacientes com diagnóstico de CMT1A foram avaliados de setembro 2018 a outubro de 2019, e outras causas de neuropatia foram excluídas. As seguintes ferramentas foram utilizadas para avaliar a dor: avaliação neurológica, LANSS, DN4, avaliação clínica, EVA, CMTNS2 e SF-36. A análise estatística foi realizada pelo teste de análise de prevalência, bem como pelos testes T, do qui-quadrado e rô de Sperman.

**Resultados:**

A prevalência de dor foi de 84,2% na amostra do estudo, com intensidade moderada e características nociceptivas de acordo com a escala LANSS (75%) e a avaliação clínica (50%), diferentemente da escala DN4, que encontrou dor neuropática na maioria dos pacientes (56,2%). Dor mista também foi verificada em 43,7% dos pacientes, de acordo com os critérios clínicos. Houve significância estatística da correlação entre a intensidade da dor e o SF-36, demonstrando que quanto menor a dor, menor o comprometimento em todos os domínios.

**Conclusão:**

A dor é um sintoma prevalente e relevante na CMT1A, com intensidade moderada e características nociceptivas de acordo com duas ferramentas, mas dor neuropática também está presente, e ainda pode haver padrão misto de dor. A correlação da dor com SF-36 sugere que o alívio da dor pode proporcionar melhorias na qualidade de vida desses indivíduos.

## INTRODUCTION

Charcot-Marie-Tooth disease (CMT) is a hereditary peripheral neuropathy that has heterogeneous genetic and clinical expression, Its prevalence is at least 1 in 2,500 individuals or 1 in 1,214, depending on the ethnicity and evaluation method. The classic phenotype of CMT disease includes normal initial development, followed by gradual distal weakness and atrophy and sensory loss arising between the first and second decades of life, with a reduction in deep tendon reflexes and skeletal deformities in the feet^1^^,^^2^^,^^3^^,^^4^.

CMT type 1A (CMT1A) is the most common hereditary neuropathy and accounts for 40 to 60% of all cases with a confirmed molecular diagnosis. It is caused by a duplication on chromosome 17p11.2, which encodes the peripheral myelin protein gene (PMP22), with a dominant pattern of inheritance. It is a demyelinating form of CMT, presenting with a homogeneous reduction in the motor conduction velocity and a significant reduction in amplitude due to secondary axonal loss^1^^,^^5^^,^^6^^,^^7^^,^^8^. Despite the growing number of studies on possible treatments, there is still no treatment available for CMT1A.

Sensory symptoms, and in particular pain, are relevant in relation to quality of life and can represent an important issue in management of CMT disease^5^^,^^9^^,^^10^^,^^11^. Despite being underdiagnosed, pain is a common complaint in these patients. However, few studies have evaluated the real pathophysiology of this symptom and there are, to date, no studies that have comprehensively evaluated its characteristics and classification^9^^,^^10^^,^^11^^,^^12^.

The aims of this study were to investigate the prevalence and characteristics of pain in a sample of patients with CMT1A and to correlate the intensity and type of pain with clinical findings, in order to ascertain its impact on quality of life.

## METHODS

This was an observational, cross-sectional and descriptive study.

### Patients

A convenience sample of 19 patients with a molecular diagnosis of CMT1A participated in this study. They were recruited from a larger study (n = 48) on patients with CMT disease that was carried out at the Clinical Research Unit in Neurology (NeuroUPC) of the Universidade Federal Fluminense (UFF), Brazil, from September 2018 to October 2019, after approval by the UFF research ethics committee and after the participating patients or their guardians had signed a free and informed consent statement.

Patients with other causes of polyneuropathy (diabetes, B12 hypovitaminosis or hypothyroidism) were excluded from this study.

### Assessment

All the patients underwent a neurological evaluation consisting of an anamnesis and a physical examination, and genetic tests were considered to confirm the CMT1A disease. All the patients underwent laboratory tests and nerve conduction studies.

Specific questionnaires were used to assess the following variables: pain intensity, using a visual analogue scale (VAS); type of pain, using the Leeds Assessment of Neuropathic Symptoms and Signs (LANSS) and the Douler Neuropathique 4 (DN4); impact on quality of life, using the SF-36 scale; and severity of CMT disease, using the Charcot-Marie-Tooth Neuropathy Score 2 (CMTNS2).

The VAS consisted of a horizontal line of 10 centimeters, on which 0 represented 'no pain' and 10 represented 'unbearable pain'. The scores obtained were categorized as follows: 1 to 3 as mild pain, 3 to 8 as moderate pain and 8 to 10 as severe pain^13^.

We used two different instruments to assess the type of pain, with the objective of obtaining greater data reliability. In this regard, it is important to remember that the sensitivity and specificity of the scales used predict possible failures: LANSS presents 85% sensitivity and 80% specificity; while DN4 presents 100% sensitivity and 93.2% specificity in its version validated for Portuguese^14^^,^^15^^,^^16^^,^^17^^,^^18^. LANSS aims to differentiate neuropathic pain from nociceptive pain and is based on analysis on assessments of sensory descriptors and sensory deficits^14^^,^^15^^,^^16^.

From the LANSS and DN4 questionnaires, types of pain can be categorized into neuropathic or nociceptive pain. Therefore, we assessed the coefficient of correlation between these types. Since the specific scales do not include mixed pain as a separate type, we evaluated a correlation between mixed pain and the clinical evaluation, according to the data collected in the anamnesis and physical examination. Hence, we were able to categorize the type of pain as neuropathic, nociceptive or mixed pain.

To this end, we used the clinical characteristics proposed by Nascimento and Schestatsky. We considered nociceptive pain to be a form of pain that was limited to the joint that was additional to mechanical pain, as pain that appeared during movement or in a static posture (standing or sitting) or that improved with rest. We also considered that nociceptive pain could be exacerbated by digital pressure and that imaging examinations at the same pain site would show myofascial or joint alterations^19^^,^^20^^,^^21^. In order to verify the characteristics of neuropathic pain, we observed whether, on physical examination, the pain pathway respected nerve territories or whether there was any change in sensitivity in the painful area. The most common descriptors of neuropathic pain were also taken into account: in addition to pain that worsened at night, spontaneous pain that was unrelated to movement or static posture^14^^,^^22^^,^^23^.

For assessment of pain and its impact on the quality of life, the SF-36 questionnaire was used, which considers eight domains of pain. The resulting values ​​range from 0 to 100, where 0 represents the greatest impact and 100 represents the least impact^24^.

The “Charcot-Marie-Tooth Neuropathy Score 2” (CMTNS2) is a validated tool that has the aim of ascertaining the impairment caused by Charcot-Marie-Tooth disease through assessing signs and symptoms, along with neurophysiological data. It uses an index that guides attribution of a score, which classifies the patients' involvement as mild (≤ 10), moderate (11-20) or severe (> 20)^25^.

Regarding the patients’ degree of muscle strength, we categorized the presence of muscle weakness as a Medical Research Council (MRC) score < 5.

### Statistical analysis and data processing

The data were collected and stored in a source document and database. Central trend measurements were used to characterize the values, together with the basic demographic data.

Independent samples, t tests and chi-square tests were performed to correlate the pain location with muscle weakness, type of pain and orthopedic abnormalities/atrophy.

## RESULTS

A description of the study population is shown in Table 1.

**Table 1.** Demographic data of patients with Charcot-Marie-Tooth disease type 1A.

	TOTAL	SD
n	19	
Age (years)	40.8 (14-77)	± 15.6
Female sex	14 (73.6%)	-
In employment	12 (63.1%)	-
Years since symptom onset	24.2 (0-53)	± 17.6
Usage of pain medication	4 (21 %)	-
Corrective surgeries	2 (10.4%)	-
Use of gait assistive device	1 (5.2%)	-
Wheelchair	0	-
Orthosis	3 (15.7%)	-
History of fractures	6 (31.5%)	-
Scoliosis	3 (15.7%)	-
Pes cavus	15 (78.9%)	-
Hammer toes	9 (47.3%)	-
Peroneal atrophy	8 (42.1%)	-
Hand atrophy	10 (52.6%)	-
Tremor	10 (52.6%)	-
CMTNS2		
Mild	4 (21%)	-
Moderate	12 (63.1%)	-
Severe	3 (15.7%)	-

SD: standard deviation.

**Figure 1.**
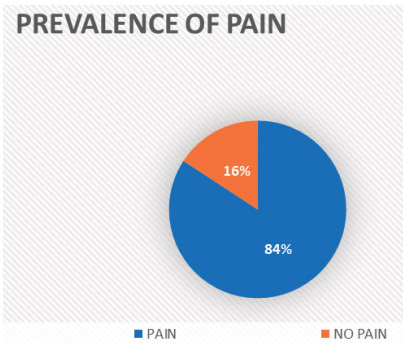
Prevalence of pain complaints among patients with Charcot-Marie-Tooth disease type 1A.

Pain was a symptom in 16 out of the 19 patients (84.2%), as shown in Figure 1. The mean age of the pain group was 41.9 ± 16.0 years, and the mean duration of symptoms since the onset of the disease was 22.8 ± 18.9 years.

The pain intensity according to the VAS scale was moderate (56.2%), with a mean score of 3.56 ± 2.98.

According to the LANSS questionnaire, 75% of patients (n = 12) had nociceptive pain and 4 patients had neuropathic pain (25%), with a mean score of 7.13 ± 5.61.

Differently, in the DN4 questionnaire, a majority of the patients (9 individuals; 56.2%) had neuropathic pain (DN4 ≥ 4), while 7 patients (43.7%) had nociceptive pain, with a mean score of 3.56 ± 2.60.

Correlation of the data from the specific scales with the data obtained through clinical evaluations showed that one patient whose characteristics of pain ascertained from the anamnesis and physical examination were entirely nociceptive, was scored as presenting neuropathic pain in both questionnaires (LANSS and DN4), thus representing a false positive. This patient had joint pain in the knees, and an imaging examination showed joint edema in both knees.

In addition, we found that, according to the clinical evaluation, seven patients (43.7%) had mixed pain, while eight patients had exclusively nociceptive pain (50%) and one patient had exclusively neuropathic pain, representing 6.2%. We considered these results, obtained through clinical evaluation, to be the standard for defining the type of pain (Table 2).

**Table 2.** Relationship between pain location and type of pain, according to the clinical evaluation, using the chi-square test.

Pain location	Frequency (n = 16)	Type of pain			p-value
		Neuropathic (n = 1)	Nociceptive (n = 8)	Mixed (n = 7)	
Feet	10 (62.5%)	1	2	7	0.008
Legs	9 (56.2%)	0	5	4	0.493
Knees	6 (37.5%)	0	3	3	0.710
Low back	5 (31.2%)	0	2	3	0.595
Neck	4 (25%)	0	2	2	0.827
Chest	3 (18.7%)	0	2	1	0.768
Hips	3 (18.7%)	0	1	2	0.644
Hands	3 (18.7%)	0	2	2	0.827
Head	2 (12.5%)	1	1	0	0.018
Forearm	2 (12.5%)	0	1	2	0.644

Pain correlations with the VAS scale and the SF-36 domains showed a significant negative correlation, thus demonstrating that the greater the intensity of pain was, the greater the impairment in quality of life also was, in all domains (Table 3).

**Table 3.** Correlations between pain intensity on visual analogue scale (VAS) and quality-of-life descriptors (SF-36).

Domain	Rho	p-value
General health	-0.531	0.034
Limitation due to physical aspects	-0.721	0.002
Functional capacity	-0.684	0.003
Mental health	-0.804	<0.01
Vitality	-0.762	0.001
Social aspects	-0.674	0.004
Bodily pain	-0.638	0.008
Limitation due to emotional aspects	-0.543	0.030

Orthopedic abnormalities and atrophy were observed in 14 patients with pain (87.5%): pes cavus in 13 patients (81.2%), hammer toes in seven (43.7%), peroneal atrophy in six (37.5%) and hand atrophy in eight (50.0%). There was no relationship with the type of pain, according to the relationship between the clinical evaluation and specific scales.

Most of the CMT patients with pain complaints had moderate disease severity according to the CMTNS2 severity scale (n = 9; 56.25%), while four patients were ranked as mild (25%) and three as severe (18.7%), with no significant correlation between the severity of the disease and the intensity and type of pain.

## DISCUSSION

The prevalence of pain was previously ascertained in a few studies that had proposed to evaluate this subject. In the present study, the prevalence of pain was 84.2%. Comparatively, in another study conducted on a sample of patients with CMT1A, the prevalence of pain was 88%^25^. An Italian study observed that 56% of individuals with CMT had pain, and that pain was the main symptom in 15 (28.8%) of these individuals^26^.

Regarding the intensity of pain, 56.2% of our cases had moderate pain. Comparatively, another study observed that 79.4% of the patients presented this result^8^. We believe that one possible reason why these patients’ pain was of moderate level may have been the slow evolution of their neuropathy, which may have enabled these individuals to use adaptive strategies for their activities of daily living.

In our study, the pain was assessed as having predominantly nociceptive characteristics according to the LANSS scale (75%). This differed from the assessment using the DN4 scale, which found neuropathic pain in a majority of the patients (56.25%).

In comparing our results with those from a study conducted in Italy and the United Kingdom, which evaluated the characteristics of pain in 49 patients with Charcot-Marie-Tooth disease type 1A^25^, using the LANSS questionnaire, similar proportions were found in that study, such that only 18% of their patients had neuropathic pain. In another study that evaluated neuropathic pain^24^ using the DN4 pain assessment tool, 62.5% of their patients presented this type of pain, compared with 56.2% of the sample in our study.

We can consider that the need to evaluate mixed pain is a limitation of the scales used, since their outcomes only allow dichotomous results for characterizing the type of pain. Therefore, we saw a need to corroborate these results by using those obtained through clinical evaluation, given that clinical results can be considered “sovereign”. Among our patients, a majority showed exclusively nociceptive characteristics (50.0%; n = 8), while seven patients had mixed pain (43.7%) and one patient had exclusively neuropathic pain (6.2%).

We correlated the other SF-36 domains with pain intensity and found statistically significant negative correlations for all domains, thus corroborating the hypothesis that the greater the pain intensity was, the greater the impact was on patients' quality of life. The subjectivity of pain symptoms still poses a great challenge with regard to evaluating and understanding pain, but investigation of pain symptoms is necessary because of their direct relationship with predictors of quality of life.

The pain locations most frequently found in the present study corroborate the hypothesis that pain in CMT disease is distal, peripheral and symmetrical. Pain was an important symptom in our study, albeit only of moderate intensity. Feet, legs and knees were the most frequent pain location and the greater the pain was, the greater the impact was in patient´s quality of life. Regarding its characteristics, the pain was of nociceptive origin in most of the individuals, according to two assessment methods (LANSS and clinical assessment), although the results showed that neuropathic pain was also present. There may also have been a mixed pattern of pain, which could only be verified through clinical evaluation.

So far, this is the largest study to have evaluated the pain of patients with Charcot-Marie-Tooth disease in Brazil. However, further research with larger samples or new tools for assessing pain is necessary in order to continue to investigate the pathophysiological aspects of pain and possible preventive measures for biomechanical changes that may cause or perpetuate pain, given its impact on the quality of life.
